# Feasibility of Co-Targeting HER3 and EpCAM Using Seribantumab and DARPin–Toxin Fusion in a Pancreatic Cancer Xenograft Model

**DOI:** 10.3390/ijms24032838

**Published:** 2023-02-02

**Authors:** Tianqi Xu, Alexey Schulga, Elena Konovalova, Sara S. Rinne, Hongchao Zhang, Olga Vorontsova, Anna Orlova, Sergey M. Deyev, Vladimir Tolmachev, Anzhelika Vorobyeva

**Affiliations:** 1 Department of Immunology, Genetics and Pathology, Uppsala University, 751 85 Uppsala, Sweden; 2 Research Centrum for Oncotheranostics, Research School of Chemistry and Applied Biomedical Sciences, National Research Tomsk Polytechnic University, Tomsk 634050, Russia; 3 Molecular Immunology Laboratory, Shemyakin-Ovchinnikov Institute of Bioorganic Chemistry, Russian Academy of Sciences, Moscow 117997, Russia; 4 Department of Medicinal Chemistry, Uppsala University, 751 23 Uppsala, Sweden; 5 Science for Life Laboratory, Uppsala University, 751 23 Uppsala, Sweden; 6 Bio-Nanophotonic Laboratory, Institute of Engineering Physics for Biomedicine (PhysBio), National Research Nuclear University ‘MEPhI’, Moscow 115409, Russia; 7 Center of Biomedical Engineering, Sechenov University, Moscow 119991, Russia

**Keywords:** pancreatic cancer, targeted therapy, HER3, MM-121, DARPin, EpCAM, pseudomonas exotoxin A, combination treatment

## Abstract

Pancreatic cancer (PC) is one of the most aggressive malignancies. A combination of targeted therapies could increase the therapeutic efficacy in tumors with heterogeneous target expression. Overexpression of the human epidermal growth factor receptor type 3 (HER3) and the epithelial cell adhesion molecule (EpCAM) in up to 40% and 30% of PCs, respectively, is associated with poor prognosis and highlights the relevance of these targets. Designed ankyrin repeat protein (DARPin) Ec1 fused with the low immunogenic bacterial toxin LoPE provides specific and potent cytotoxicity against EpCAM-expressing cancer cells. Here, we investigated whether the co-targeting of HER3 using the monoclonal antibody seribantumab (MM-121) and of EpCAM using Ec1–LoPE would improve the therapeutic efficacy in comparison to the individual agents. Radiolabeled ^99m^Tc(CO)_3_-Ec1–LoPE showed specific binding with rapid internalization in EpCAM-expressing PC cells. MM-121 did not interfere with the binding of Ec1–LoPE to EpCAM. Evaluation of cytotoxicity indicated synergism between Ec1–LoPE and MM-121 in vitro. An experimental therapy study using Ec1–LoPE and MM-121 in mice bearing EpCAM- and HER3-expressing BxPC3 xenografts demonstrated the feasibility of the therapy. Further development of the co-targeting approach using HER3 and EpCAM could therefore be justified.

## 1. Introduction

Pancreatic cancer (PC) is one of the most aggressive malignancies with a rapidly growing incidence and high mortality [1,2]. PC ranks in the top ten causes of cancer-related deaths worldwide, particularly in developed countries [3]. The symptoms of PC are commonly insidious at an early stage, leading to the majority of patients being diagnosed with advanced disease when the cancer has already metastasized [4]. Due to the lack of early detection, PC is characterized by a poor prognosis [5,6]. Patients diagnosed with PC have a median overall survival of fewer than six months and a five-year survival rate of 8–12% [5].

Conventional approaches to the treatment of pancreatic cancer include surgical resection in combination with chemotherapy and/or radiation therapy [7]. However, up to 80% of patients are not eligible for surgery due to a late diagnosis. Patients who underwent surgical resection with adjuvant therapy commonly have a recurrence with locoregional or systemic metastasis and a five-year survival rate of 20–25% [7].

Treatment of non-resectable and metastatic cancer with systemic chemotherapy has limited efficacy due to a narrow therapeutic window and toxicity to healthy tissues [7]. Targeted therapy approaches might provide more effective and safe treatment options for PC as they localize the cytotoxic effect to cancer cells and minimize general toxicity. A number of targeting agents have been evaluated for the treatment of PC, such as tyrosine kinase inhibitors (e.g., erlotinib [8]), poly(ADP-ribose)polymerase (PARP) inhibitors (e.g., olaparib [9]), and monoclonal antibodies (mAbs; e.g., bevacizumab targeting vascular endothelial growth factor (VEGF) [10] and cetuximab targeting epidermal growth factor receptor (EGFR) [11]).

Despite a large number of clinical trials aiming to identify a single-agent treatment for advanced PC [12], the only chemotherapeutic agent shown to improve survival was gemcitabine [13], which remains the current standard of care [7]. A combination consisting of leucovorin, fluorouracil, irinotecan, and oxaliplatin (FOLFIRINOX) was found to be more effective than gemcitabine alone for the treatment of metastatic disease; however, the high toxicity of this regimen limits its use [14,15].

One advantage of combining agents that bind to different molecular targets is an increased cytotoxic effect to cancer cells with a potential reduction in off-target toxicities. Since the expression of molecular targets in tumors is often heterogeneous, combination treatment might reach a larger fraction of cells compared to monotherapy. Furthermore, by targeting different mechanisms, combination treatments might prevent the development of resistance, which is commonly observed after several cycles of conventional chemotherapy.

One potential molecular target in PC is human epidermal growth factor receptor 3 (HER3). HER3 is a transmembrane protein that belongs to the receptor tyrosine kinases family. It plays a critical role in cell regulation, including cellular proliferation, differentiation, and survival [16]. Overexpression of HER3 was identified in various malignant tumor types, such as breast, gastric, colorectal, prostate, lung, ovarian, and pancreatic cancers [17,18,19,20,21,22]. HER3 overexpression is found in up to 40% of PCs and is associated with a more advanced stage, indicating a poor prognosis [23]. In addition, HER3 is involved in the acquired resistance to anti-EGFR treatment in patients with PC [24].

Seribantumab (MM-121) is a fully human IgG2 mAb that binds specifically to HER3 with high affinity (picomolar), functioning as an antagonist and blocking ligand-dependent activation of HER3 [25]. MM-121 was studied in clinical trials for several types of cancer. In patients with breast cancer, MM-121 did not increase progression-free survival and the trial was terminated in the late phase [26]. The lack of precise selection of patients for the trial may be one of the reasons behind its failure [25]. Due to the heterogeneity of HER3 expression, it is necessary to select potential responders with HER3-overexpressing tumors for MM-121 treatment [27,28]. The development of HER3 imaging agents using, for example, radiolabeled antibodies, antibody fragments, nanobodies, short peptides, and affibody molecules, would be desirable to select patients for HER3-targeted therapy [29,30,31,32,33,34,35,36].

The epithelial cell adhesion molecule (EpCAM) is another attractive target for PC treatment. It is a transmembrane glycoprotein participating in cell adhesion, proliferation, migration, and invasion [37,38]. EpCAM has an increased level of expression on epithelial tumor cells, such as adenocarcinomas of the colon, stomach, pancreas, and prostate [39,40]. In PC, 23–37% of patients present with EpCAM overexpression, which is associated with a poor prognosis and is correlated with shorter overall survival [40]. EpCAM consists of an extracellular part with an epidermal growth factor-like domain and a human thyroglobulin-like domain, a single transmembrane helix, and a short cytoplasmic tail [41]. The binding of EpCAM-targeting agents to the extracellular domain triggers internalization by endocytosis. This mechanism allows for targeted drug delivery with a therapeutic purpose [42,43,44]. Immunotoxins, which consist of an anti-EpCAM agent conjugated to a cytotoxic payload, were studied in recent years and many EpCAM-targeting immunotoxins reached clinical trials [45]. One example is MOC31PE, where the antibody MOC31 was conjugated to the cytotoxic agent Pseudomonas exotoxin A (PE). The MOC31PE treatment significantly increased survival in patients with colorectal peritoneal metastasis [46].

Previously, the EpCAM-targeting toxin fusion protein Ec1–LoPE (43 kDa) was studied in prostate cancer cells, ovarian cancer cells, and breast cancer cells for its cytotoxic effect [47,48,49]. Ec1–LoPE consists of the EpCAM-targeting agent Ec1 (18 kDa), a designed ankyrin repeat protein (DARPin), fused with the cytotoxic agent LoPE (25 kDa) at the C-terminus. DARPins are a type of engineered scaffold protein (ESP) with a molecular weight from 14 to 18 kDa. Similar to other ESPs, DARPins have several advantages over antibodies and antibody fragments that facilitate the delivery of a cytotoxic drug to tumors. First, the small size of DARPins can promote the delivery efficiency of the cytotoxic payload due to superior extravasation and tumor penetration. Second, the recombinant production of ESPs provides higher yields with lower manufacturing costs compared to antibodies [50,51,52]. Ec1 has shown high affinity to EpCAM with an equilibrium dissociation constant (K_D_) in the picomolar range [53]. The LoPE toxin (25 kDa) is derived from Pseudomonas exotoxin A (PE), with silenced human and murine B cell recognition epitopes [54]. This modification reduces its immunogenicity and general toxicity [5,47].

The inter- and intra-tumoral heterogeneity of PC makes combination targeted therapy an attractive strategy [5]. Co-treatment using drugs with different action modes could help to overcome the heterogeneity of target expression in tumors, spread the toxicity to critical organs, and prevent the development of resistance [55]. In a previous study, co-treatment with Ec1–LoPE and the anti-HER2 monoclonal antibody trastuzumab delayed tumor growth and significantly prolonged survival of mice bearing EpCAM- and HER2-expressing ovarian cancer SKOV3 xenografts compared to single treatments, while no pathological changes in normal organs, e.g., kidneys and livers, were detected [48]. MM-121 demonstrated therapeutic efficacy in a preclinical study in pancreatic BxPC3 xenografts [36].

The goal of this study was to test the hypothesis that the combined treatment using the EpCAM-targeting Ec1–LoPE toxin and the HER3-targeting monoclonal antibody MM-121 would increase the efficacy of experimental therapy in a pancreatic cancer model. To test this hypothesis, the binding specificity of Ec1–LoPE to two EpCAM-expressing pancreatic cancer cell lines, BxPC3 and Panc-1, was tested and the affinity of Ec1–LoPE was measured. We evaluated if co-treatment with monoclonal antibody MM-121 would interfere with the binding of Ec1–LoPE to BxPC3 and Panc-1 cells in vitro. The rate of Ec1–LoPE internalization by BxPC3 and Panc-1 cells in vitro was measured. To facilitate a quantitative evaluation in these experiments, Ec1–LoPE was site-specifically labeled with the radionuclide ^99m^Tc. Half-value growth inhibition of BxPC3 and Panc-1 cells by Ec1–LoPE was measured. A possible synergistic effect of in vitro treatment of BxPC3 cells (expressing both EpCAM and HER3) using Ec1–LoPE and monoclonal antibody MM-121 was evaluated. The effect of monotherapies using MM-121 and Ec1–LoPE and a combination of these therapies was compared in vivo in mice bearing BxPC3 xenografts.

## 2. Results

### 2.1. Radiolabeling

Ec1–LoPE was radiolabeled with tricarbonyl technetium-99m with a radiochemical yield of 48 ± 6% (n = 4). Purification using NAP-5 size-exclusion column provided a radiochemical purity of 99 ± 1% (n = 4).

### 2.2. In Vitro Specificity and Cross-Blocking Study

**Figure 1:**
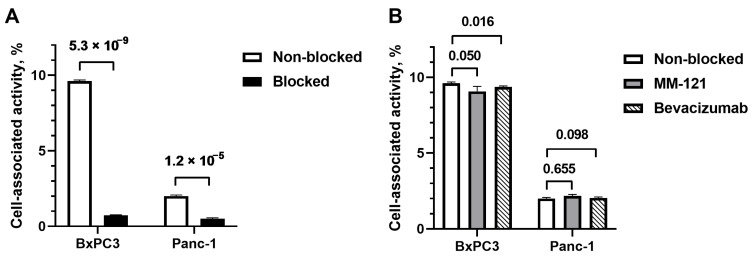
In vitro binding of [^99m^Tc]Tc(CO)_3_-Ec1–LoPE in EpCAM-expressing BxPC3 and Panc-1 cells. (**A**) Specificity of [^99m^Tc]Tc(CO)_3_-Ec1–LoPE binding; 100-fold molar excess of DARPin Ec1 (200 nM) was used to block EpCAM receptors. (**B**) Cross-blocking study of [^99m^Tc]Tc(CO)_3_-Ec1–LoPE by a 100-fold molar excess of anti-HER3 antibody MM-121 and anti-VEGF-A antibody bevacizumab as a control. The final concentration of the radiolabeled [^99m^Tc]Tc(CO)_3_-Ec1–LoPE was 2 nM. The data are presented as average ± standard deviation (SD) (n = 3). *p* values from unpaired *t*-tests are provided to demonstrate differences between activity uptake in non-blocked and blocked groups.

The binding specificity of [^99m^Tc]Tc(CO)_3_-Ec1–LoPE to EpCAM was investigated using EpCAM-expressing BxPC3 and Panc-1 pancreatic cancer cells. The binding of radioactivity to both cell lines was significantly (*p* < 0.05) decreased when a large excess of non-labeled Ec1 was added (Figure 1A), indicating that the binding of [^99m^Tc]Tc(CO)_3_-Ec1–LoPE to both cell lines was EpCAM-specific.

To investigate whether MM-121 interfered with the binding of [^99m^Tc]Tc(CO)_3_-Ec1–LoPE to EpCAM, an excess of MM-121 was added to the cells before the addition of [^99m^Tc]Tc(CO)_3_-Ec1–LoPE. As an IgG antibody control, an excess of bevacizumab, which does not bind to any receptor in the HER family, was used in the third group. Compared to the non-blocked group, the uptake of activity in the MM-121-treated groups did not differ significantly (*p* > 0.05) in both cell lines (Figure 1B). There was a minor but significant (*p* = 0.016) decrease of cell-associated activity in the bevacizumab-treated BxPC3 cells; however, this was not observed in Panc-1 cells (*p* = 0.098). These results showed that MM-121 did not decrease the binding of [^99m^Tc]Tc(CO)_3_-Ec1–LoPE to EpCAM.

### 2.3. In Vitro Cellular Processing and Internalization Study

**Figure 2:**
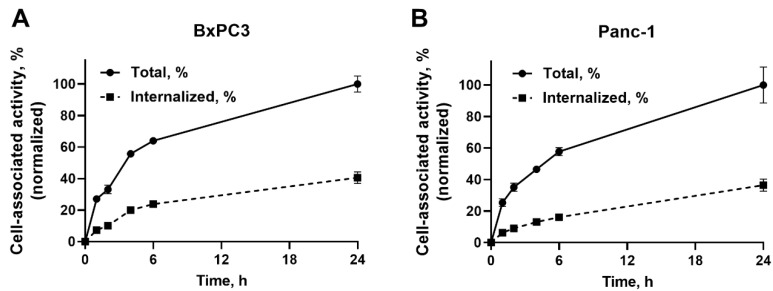
In vitro cellular processing and internalization of [^99m^Tc]Tc(CO)_3_-Ec1–LoPE in EpCAM-expressing BxPC3 and Panc-1 cells. Data were normalized to the value of total cell-associated activity at 24 h taken as 100%. The data are presented as the average ± SD (n = 3).

To evaluate the internalization and cellular processing rate, [^99m^Tc]Tc(CO)_3_-Ec1–LoPE was continuously incubated with BxPC3 and Panc-1 cells for 1, 2, 4, 6, and 24 h (Figure 2). The cell-associated activity steadily increased over time and reached maximum by 24 h in both cell lines. The internalized fractions were 41 ± 4% in BxPC3 cells and 37 ± 4% in Panc-1 cells by 24 h.

### 2.4. Real-Time Binding of [^99m^Tc]Tc(CO)_3_-Ec1–LoPE to BxPC3 Cells

The real-time interaction of [^99m^Tc]Tc(CO)_3_-Ec1–LoPE with EpCAM-expressing BxPC3 cells was investigated using a LigandTracer Yellow instrument. According to the Interaction Map analysis, two major interactions were observed, with the first interaction of 1.03 ± 0.05 nM (31 ± 8% weight), and a second interaction of 27 ± 9 nM (27 ± 9% weight) (Table 1).

### 2.5. Cytotoxicity

**Figure 3:**
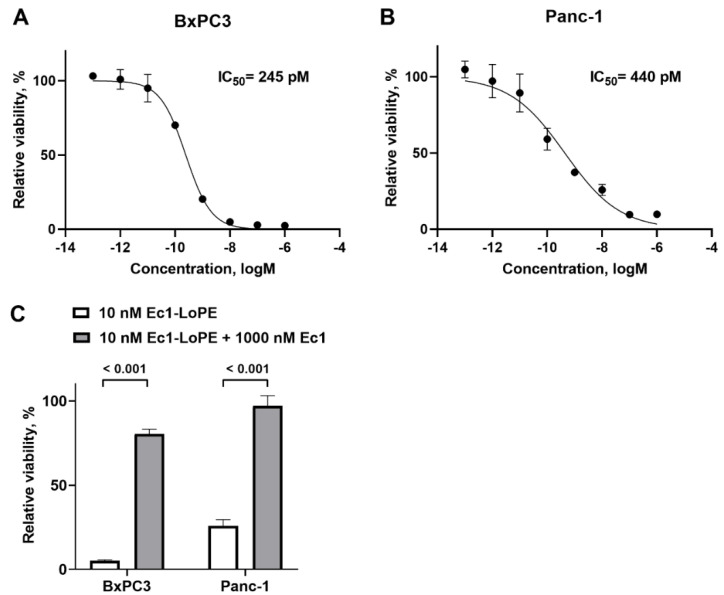
The cytotoxic effect of Ec1–LoPE in EpCAM-expressing (**A**) BxPC3 and (**B**) Panc-1 cells in vitro. (**C**) Specificity of Ec1–LoPE action in BxPC3 and Panc-1 cells. The viability of cells grown in medium (no Ec1–LoPE added) was used as 100% viability control. Relative viability (the *y*-axis) was plotted versus concentrations of Ec1–LoPE (the *x*-axis) to derive the viability curve. The data are presented as average ± SD of five samples.

The cytotoxic effect of Ec1–LoPE was evaluated in EpCAM-expressing BxPC3 and Panc-1 cell lines by incubating the cells with a series of Ec1–LoPE concentrations and measuring cell viability. Ec1–LoPE demonstrated a dose-dependent cytotoxic effect with IC_50_ values of 245 pM in BxPC3 cells and 440 pM in Panc-1 cells (Figure 3A,B). The cytotoxic action of Ec1–LoPE was significantly (*p* < 0.001) reduced in both cell lines when the cells were incubated with Ec1 before the addition of Ec1–LoPE to block binding of the targeting toxin (Figure 3C), which suggested an EpCAM-dependent action of Ec1–LoPE.

**Figure 4:**
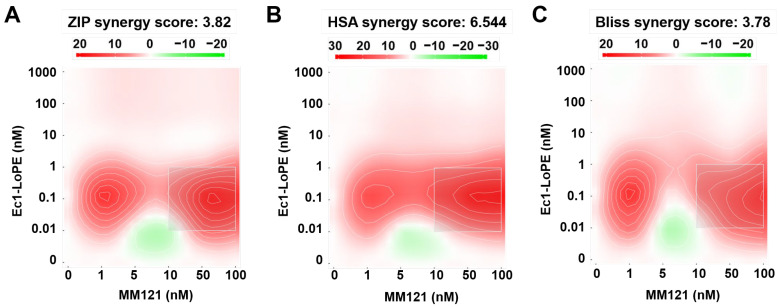
Heat map of synergistic effects. The viability of BxPC3 cells after incubation with Ec1–LoPE (0–1000 nM) and MM-121 (0–100 nM). Graphs display Ec1–LoPE concentration (nM) on the *y*-axis and MM-121 concentration (nM) on the *x*-axis. Values equal to zero (white area) are counted as an additive effect. Values above zero (white to red area) are counted as a synergistic effect, and values below zero (white to green area) are counted as an antagonistic effect.

The effect of Ec1–LoPE in combination with MM-121 on cell viability was evaluated in EpCAM- and HER3-expressing BxPC3 cells. The viability data were analyzed using SynergyFinder 3.0 software and the synergy score was calculated using three different reference models. The resulting synergy scores for all the models were above zero (3.8 for the ZIP model, 6.5 for the HSA model, and 7.3 for the Bliss model), which indicated synergism between Ec1–LoPE and MM-121 (Figure 4). The most synergistic areas in all three models corresponded to 0.01-1 nM concentrations of Ec1–LoPE and 10–100 nM concentrations of MM-121.

### 2.6. Experimental Therapy Using Ec1–LoPE and MM-121

**Figure 5:**
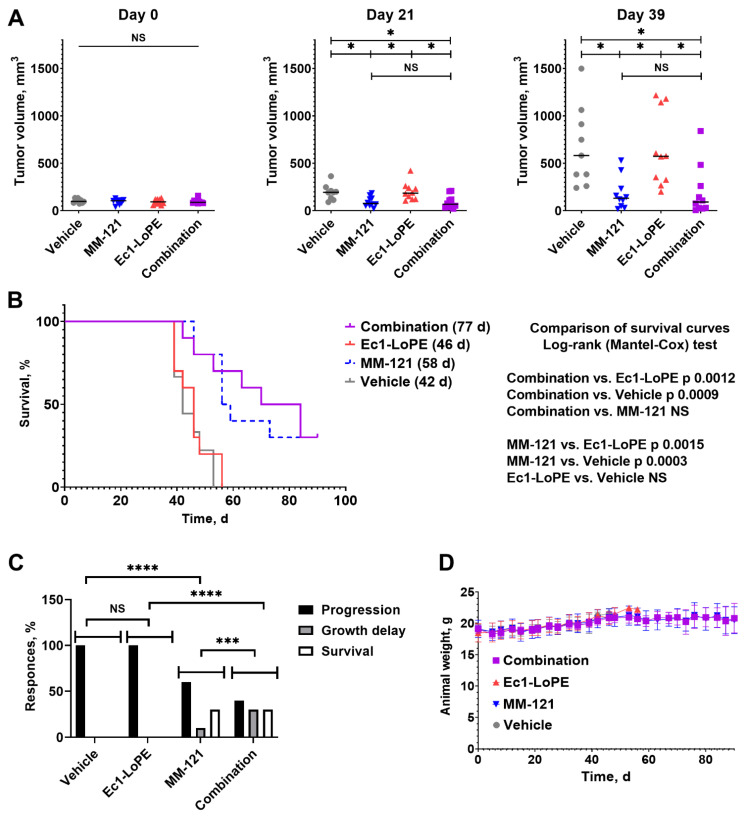
Experimental therapy in BALB/c nu/nu mice bearing BxPC3 xenografts receiving Ec1–LoPE and MM-121 combination treatment, Ec1–LoPE single treatment, MM-121 single treatment, or vehicle (0.9% NaCl). (**A**) Comparison of average tumor volumes at the start of the experiment (day 0), at the start of the second treatment cycle using Ec1–LoPE (day 21), and eight days after the last treatment cycle (day 39). NS corresponds to no statistically significant difference (*p* > 0.05), * corresponds to *p* < 0.05 (one-way ANOVA with Bonferroni correction). (**B**) Survival of the mice during experimental therapy. (**C**) Therapy outcomes for different treatment groups. The outputs categories were defined as progression (exponential tumor growth), growth delay (initial growth suppression followed by exponential tumor growth), and survival (animals did not reach a humane endpoint at the study termination). The difference between groups was determined using the chi-square test. NS corresponds to no statistically significant difference (*p* > 0.05), *** corresponds to *p* < 0.001, **** corresponds to *p* < 0.0001. (**D**) Average animal weight in each group during the therapy experiment. The data are presented as an average value ± SD.

The efficiency of combination treatment using Ec1–LoPE and MM-121 was compared to the efficiency of monotherapies using Ec1–LoPE and MM-121 and to the vehicle control in female BALB/c nu/nu mice bearing BxPC3 xenografts. There were no statistically significant (*p* > 0.05) differences in average tumor volume between the groups at the start of the experiment (Figure 5A). On day 21, after four injections of Ec1–LoPE and six injections of MM-121, the tumors in the combination therapy group (93 ± 68 mm^3^) and MM-121 monotherapy group (95 ± 53 mm^3^) were significantly (*p* < 0.05) smaller than tumors in the Ec1–LoPE monotherapy group (203 ± 94 mm^3^) and the control group (194 ± 81 mm^3^). The same trend of significant (*p* < 0.05) differences between the groups was observed until day 39 when the first five mice were euthanized in the control group and Ec1–LoPE monotherapy group.

The monotherapy with Ec1–LoPE had no measurable effect on the tumor growth in comparison with the vehicle-treated control (Figure 5B). The tumor doubling time in the Ec1–LoPE monotherapy group (9 ± 3 days) did not differ from the doubling time of the control group (10 ± 4 days). The median survival of mice in these groups was similar, 42 and 46 days, for animals treated with vehicle and Ec1–LoPE monotherapy, respectively (Figure 5B). The median survival of mice in the combination group was 77 days and in the MM-121 monotherapy group was 58 days; however, no statistically significant differences (*p* > 0.05, log-rank (Mantel-Cox) test) were found between the survival curves of mice in the combination group and MM-121-treated groups.

**Figure 6:**
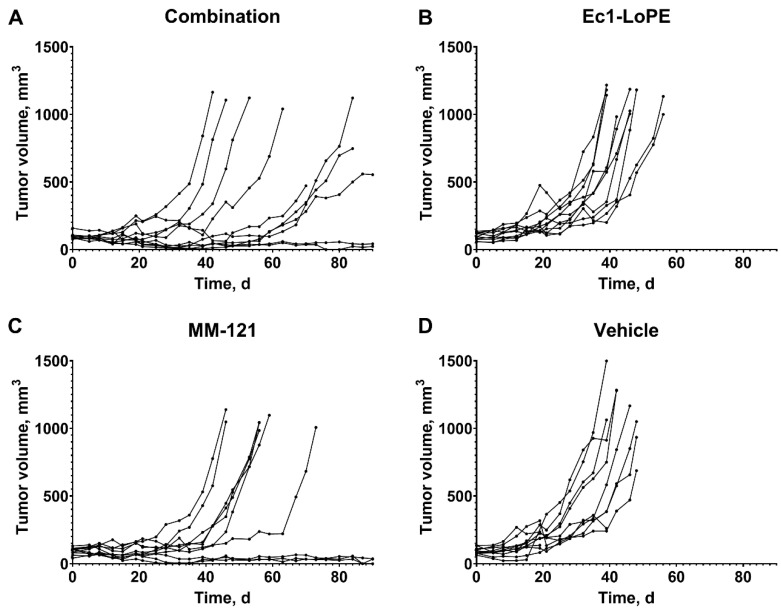
Individual tumor volume growth curves in BALB/c nu/nu mice bearing BxPC3 xenografts from four groups receiving: (**A**) intravenous (i.v.) injections of Ec1–LoPE and intraperitoneal (i.p.) injections of MM-121; (**B**) i.v. injections of Ec1–LoPE; (**C**) i.p. injections of MM-121; and (**D**) i.v. injections of vehicle (0.9% NaCl). Mice were euthanized when the xenograft volume exceeded 1000 mm^3^, bleeding ulcers on xenografts were observed, or over 10% weight loss was detected within one week.

All mice in in the vehicle and Ec1–LoPE-treated groups had exponential tumor growth (progression) (Figure 5C, Figure 6 and Figure S3, Table S1). The therapy outcomes in the groups treated with MM-121 monotherapy or the combination of MM-121 and Ec1–LoPE were different. There were some tumors, which did not respond to therapy (six mice treated with MM-121 and four mice treated with the combination), with a growth rate similar to the growth rate in the vehicle control group (doubling time 9 ± 1 days for both treatment groups). In some animals from these groups (four mice in the combination therapy and one mouse in the MM-121 monotherapy group), the tumor growth was initially suppressed but resumed after day 50. The growth of some tumors in these groups was suppressed and did not resume during the observation period. While the monotherapy using Ec1–LoPE was inefficient according to the chi-square contingency test, adding Ec1–LoPE to MM-121 potentiated it, making the combination therapy outcome significantly better than the outcome of the MM-121 monotherapy (Figure 5C).

All treatment regimens were well tolerated by mice with no observable side effects. No tendency for weight loss was observed in any of the groups. The differences in average animal weight between the groups were within the standard deviation (Figure 5D). During the histopathological examination of the liver and kidneys from mice treated with Ec1–LoPE, MM-121, or their combination, no lesions were found that could suggest toxicity of the treatment regimens (Figures S1 and S2).

## 3. Discussion

The combination of targeted therapeutics has the potential to reduce toxicity to normal tissues in the treatment of pancreatic cancer [56], which may further widen a therapeutic window. Overexpression of EpCAM in multiple malignancies indicates that complementary treatment with EpCAM-targeting agents might be a universal strategy to potentiate the effect of targeted cancer therapy.

Anti-EpCAM Ec1–LoPE DARPin–toxin fusion demonstrated efficacy in several cancer models, both alone and in combination with other targeting agents [47,48,49]. Thus, its application for a therapy-resistant malignancy like pancreatic cancer might be attractive. The mechanism of the cytotoxic action of Ec1–LoPE includes binding of Ec1 to EpCAM and receptor-mediated internalization of the EpCAM-Ec1–LoPE complex inside the cell. LoPE is a de-immunized derivative of bacterial Pseudomonas aeruginosa Exotoxin A (PE toxin) [54], which contains receptor binding domain I, intracellular processing domain II, and the catalytically active domain III, which mediates toxic action. Domain III is an NAD+-diphthamide ADP-ribosyltransferase that inactivates eukaryotic elongation factor eEF2, which leads to the inhibition of protein synthesis and cell death [57]. MM-121 binds to HER3 and blocks signaling induced by the extracellular growth factors heregulin and betacellulin. This leads to inhibition of key pro-survival pathways mediated by phosphoinositide 3-kinase (PI3K) and Akt signaling and to cancer cell death [25,58]. The combination of two agents with different mechanisms of action, Ec1–LoPE with a direct cytotoxic action and MM-121 blocking key pro-survival pathways, might increase their cytotoxic effect.

It should be noted that malignant tumors constitute a very heterogeneous group of diseases. They differ in proliferation-driving mutations, patterns of molecular target expression and co-expression, and sensitivity/resistance to different therapies. It is not a given that a therapy that is effective in one type of cancer (e.g., ovarian) would be equally effective in another one (e.g., pancreatic). The feasibility of treatment should be demonstrated in each case. The selection of an appropriate model for proof-of-principle study, including the aspects of sufficient target expression, cellular processing of a targeting agent, and cytotoxicity is critical.

To quantitatively assess the targeting properties of Ec1–LoPE in vitro, the compound was site-specifically labeled with technetium-99m tricarbonyl via hexahistidine tag. This label has residualizing properties and thus permits the study of retention after internalization in cells [59]. The same labeling method was used previously and provided a stable label [48,49]. The specificity of Ec1 binding to EpCAM was previously demonstrated by several orthogonal methods [60]. In the binding specificity experiment, the binding of [^99m^Tc]Tc(CO)_3_-Ec1–LoPE to both BxPC3 and Panc-1 cells decreased significantly (*p* < 0.05) in the presence of unlabeled Ec1 when the EpCAM receptors were saturated. This indicated that the uptake of Ec1–LoPE was EpCAM-mediated (Figure 1A). The binding to BxPC3 cells was higher than the binding to Panc-1 cells. A similar pattern was observed previously for the non-LoPE-fused Ec1, which could be explained by higher EpCAM expression in BxPC3 cells than in Panc-1 cells [61]. The binding and internalization pattern of [^99m^Tc]Tc(CO)_3_-Ec1–LoPE was similar for both cell lines (Figure 2). Both cell lines internalized the targeting toxin efficiently (approximately 40% of cell-associated activity was internalized after 24 h incubation), which created an important precondition for intracellular delivery of the toxin. The higher EpCAM expression level in BxPC3 cells correlated with the higher cytotoxicity (lower IC_50_ value) of Ec1–LoPE for this cell line (Figure 3). Importantly, the prevention of Ec1–LoPE binding to both cell lines by target saturation with Ec1 (not fused with the toxin) significantly reduced cytotoxicity (Figure 3C), which demonstrated that the effect was caused by the specific toxin delivery to cells. Overall, the cytotoxicity of Ec1–LoPE for pancreatic cancer cells (IC_50_ in the range 240–440 pM) was lower compared with the cytotoxicity for prostate cancer cell lines (IC_50_ in the range 30–60 pM) [49], but higher than that for the ovarian cancer cell line SKOV-3 (500 nM) [48]. Still, the treatment of SKOV-3 xenografts using Ec1–LoPE increased the survival of mice [48]. This suggested that the in vivo experiments using Ec1–LoPE in the pancreatic cancer model were ethically justified. For the proof-of-principle study, the BxPC3 cell line was selected because it has a higher target expression level and is more sensitive to Ec1–LoPE treatment.

The binding of Ec1–LoPE to living BxPC3 cells was characterized by two interactions (Table 1), both having K_D_ values in the nanomolar range. A similar pattern was observed in our previous studies [48,49], while higher affinity was observed for Ec1 not fused to LoPE toxin in ovarian cancer cells [53]. This suggested that the fusion of LoPE to Ec1 reduced the affinity of Ec1, which might be a result of increased molecular size and associated steric hindrance in the interaction with EpCAM.

In vitro binding experiments demonstrated that incubation of the pancreatic cancer cells with MM-121 did not reduce the binding of [^99m^Tc]Tc(CO)_3_-Ec1–LoPE (Figure 1B), suggesting that the combination treatment was feasible. Further in vitro evaluation and calculations using several models suggested that an appreciable synergistic effect of the co-treatment could be expected (Figure 4).

The experimental proof-of-principle therapy was performed in two cycles using an adaptive design. Since the results of the first Ec1–LoPE treatment cycle did not show a clear anti-tumor effect (but also no signs of general toxicity), a second cycle was initiated two weeks after the first one. Still, the monotherapy using Ec1–LoPE was inefficient and the tumor growth rate in treated mice was similar to the growth rate in the control group (Figure 6B,D). Further analysis did not reveal any difference in median survival (Figure 5B) or response rate (Figure 5C). These data are somewhat different from the data obtained for ovarian cancer [48] and breast cancer models [47], where the monotherapy had a certain anti-tumor effect. It has to be noted that different models and different treatment schemes were used in our study compared to the studies by Shramova et al. [47] and Xu et al. [48]. Further investigation and optimization of the pharmacokinetic parameters of Ec1–LoPE might be necessary to improve the delivery to tumors and its anti-tumor action in the BxPC3 xenograft model. The difference between the survival curves of mice in the combination group and mice in the MM-121 monotherapy group was not statistically significant (Figure 5B). It is of interest that the combination treatment improved therapy outcome compared with the MM-121 monotherapy. The contingency analysis demonstrated a significant difference in the outcomes of these therapies (Figure 5C). These results demonstrate the feasibility of EpCAM- and HER3-targeting therapy using Ec1–LoPE and MM-121 in pancreatic cancer.

## 4. Materials and Methods

### 4.1. General

The chemicals used in the study were purchased from Sigma-Aldrich (Sweden AB, Stockholm, Sweden). Buffers were prepared using high-quality Milli-Q water.

### 4.2. Cell Culture

Human pancreatic cancer cell lines BxPC3 and Panc-1 were purchased from the American Type Culture Collection (ATCC; LGC Promochem, Borås, Sweden). Both cell lines overexpress EpCAM [62,63] and the BxPC3 cell line overexpresses HER3 [64,65]. BxPC3 cells were cultured in Roswell Park Memorial Institute (RPMI)-1640 medium (L0500, Biowest) supplemented with 2 mM L-glutamine, 10% fetal bovine serum (FBS; F7524, Sigma-Aldrich), and 1% penicillin–streptomycin (L0022, Biowest, Berlin, Germany). Panc-1 cells were cultured in Dulbecco’s Modified Eagle’s Medium (DMEM; 11594486, Thermo Fisher Scientific, Waltham, MA, USA) supplemented with 2 mM L-glutamine, 10% FBS, and 1% penicillin–streptomycin solution. Cells were cultured at 37 °C and 5% CO_2_ atmosphere unless stated otherwise. Trypsin–ethylenediaminetetraacetic acid (EDTA) solution (25200056, Thermo Fisher Scientific, Waltham, MA, USA) was used for cell detachment.

### 4.3. Protein Production

Ec1–LoPE fusion protein and DARPin Ec1 were produced as described previously [47,61]. The monoclonal antibody seribantumab (MM-121) was purchased from Merrimack Pharmaceuticals (Cambridge, MA, USA). The molecular weights of the proteins used in this study were: Ec1–LoPE 43061 Da [49], Ec1 18348 Da [61], and MM-121 143151 Da.

### 4.4. Radiolabeling

Ec1–LoPE was site-specifically labeled with technetium tricarbonyl [^99m^Tc][Tc(CO)_3_] using a C-terminal H_6_-tag as described previously [48,49]. Briefly, 500 μL (3–5 GBq) of technetium-99m pertechnetate ([^99m^Tc]TcO_4_^−^)-containing eluate from a commercial ^99^Mo/^99m^Tc generator (Mallinckrodt, Petten, The Netherlands) in sterile 0.9% NaCl was added to a CRS kit vial (PSI, Villigen, Switzerland) and incubated at 100 °C for 30 min, followed by incubation at room temperature for 10 min. Then, the [^99m^Tc][Tc(CO)_3_(H_2_O)_3_]^+^ was neutralized with 0.1 M HCl (1:2 volume ratio) (final volume 45–100 µL; 50-300 MBq) and added to Ec1–LoPE (30 µg, 25 µL in PBS) and the mixture was incubated at 40 °C for 60 min. To remove the loosely-bound technetium tricarbonyl, a 1000-fold molar excess of histidine (110 μg, 11 μL of 10 μg/μL in PBS) was added to the reaction mixture and incubated at 40 °C for 10 min. The radiolabeled compound was purified using a NAP-5 size exclusion column (Cytiva, Uppsala, Sweden), equilibrated, and eluted with PBS. The radiochemical yield and purity of [^99m^Tc]Tc(CO)_3_-Ec1–LoPE were measured by instant thin-layer chromatography (iTLC) strips (Varian, Lake Forest, CA, USA) eluted in PBS. In this system, the radiolabeled protein remained at the application point and other forms of non-bound radionuclide moved with the PBS front. The activity distribution was measured with a Storage Phosphor System (Elysia-Raytest, Bietigheim-Bissingen, Germany) and analyzed with AIDA Image Analysis software (Elysia-Raytest, Bietigheim-Bissingen, Germany).

### 4.5. In Vitro Specificity and Cross-Blocking Study

The evaluation of binding specificity of [^99m^Tc]Tc(CO)_3_-Ec1–LoPE to EpCAM-overexpressing Panc-1 and BxPC3 cells was performed as described previously [48]. Briefly, 0.8 × 10^6^ cells/well were seeded in 6-well plates one day before the experiment. The experiment was done in triplicates. On the experiment day, the medium was removed from the cells, and Ec1 (200 nM in 0.5 mL of medium) was added to one set of wells (the blocked group). The same volume of medium only was added to another set of wells (the non-blocked group). The cells were incubated at 37 °C for 60 min. Then, [^99m^Tc]Tc(CO)_3_-Ec1–LoPE (4 nM in 0.5 mL in medium) was added to each well to reach a final concentration of 2 nM, followed by incubation at 37 °C for 60 min. After incubation, the medium was collected and cells were washed with medium (1 mL), which was collected into the same tubes. To collect the cell-associated activity, the cells were incubated with 1 M NaOH (1 mL) at 37 °C for 30 min, followed by a wash with 1 M NaOH (1 mL), and the cell lysate was collected into a tube.

To evaluate the feasibility of simultaneous targeting of the EpCAM and HER3 receptors, MM-121 (200 nM in 0.5 mL of medium) was added before the addition of [^99m^Tc]Tc(CO)_3_-Ec1–LoPE and the cells were treated as described above. To investigate whether the potential cross-blocking effect of MM-121 might be caused by the non-specific interaction of an IgG scaffold, the anti-vascular endothelial growth factor A (VEGF-A) monoclonal antibody bevacizumab (200 nM) was added to another set of wells prior to the addition of [^99m^Tc]Tc(CO)_3_-Ec1–LoPE. The activity in the tubes containing medium and cells was measured using an automatic gamma spectrometer equipped with a 3-inch NaI(Tl) well detector (2480 Wizard, Wallac, Turku, Finland) and the percent of cell-associated activity was calculated. The percent of cell-associated activity was calculated as the amount of radionuclide (in counts per minute) in the tube containing cells divided by the sum of the amounts of radionuclide in the tube containing medium and the tube containing cells.

### 4.6. In Vitro Internalization Study

The cellular processing and internalization of [^99m^Tc]Tc(CO)_3_-Ec1–LoPE by BxPC3 and Panc-1 cells was studied as described previously [66]. BxPC3 and Panc-1 cells were seeded into 3 cm Petri dishes at a density of 0.8 × 10^6^ cells/well one day before the experiment. The experiment was done in triplicates. On the experiment day, the medium was removed from the cells and [^99m^Tc]Tc(CO)_3_-Ec1–LoPE (2 nM, 1 mL) was added to each well followed by incubation at 37 °C for 1, 2, 4, 6, or 24 h. At each time point, the cell medium was collected into tubes and the cells were washed with 1 mL of ice-cold PBS, which was collected. To collect the membrane-bound activity, cells were treated with 1 mL of 0.2 M glycine buffer containing 4 M urea (pH 2.0) for 5 min on ice and the solution was collected. The cells were washed once with the same solution, which was collected. To collect the internalized activity, cells were incubated 1 M NaOH at 37 °C for 20 min and treated as described above. The activity in each fraction tube was measured using the gamma spectrometer and the percent of cell-associated activity was calculated. The data were normalized to the maximum value of cell-associated activity at 24 h that was set as 100%.

### 4.7. Interaction Analysis Using Ligand Tracer

The interaction of [^99m^Tc]Tc(CO)_3_-Ec1–LoPE with living BxPC3 cells was measured using the LigandTracer Yellow instrument (Ridgeview Instruments AB, Vänge, Sweden) as described previously [48]. Cells (2 × 10^6^) were seeded to a local area of an 8.9 cm Petri dish (Nunclon TM, Roskilde, Denmark) one day before the experiment. The experiment was performed at room temperature to prevent internalization. After 30 min of background measurement, [^99m^Tc]Tc(CO)_3_-Ec1–LoPE was added (3 and 9 nM) and the measurement was performed for 90 min at every concentration. Thereafter, the medium containing the radiolabeled compound was replaced by fresh medium to measure the dissociation rate. The binding curve was analyzed by the TracerDrawer software (Ridgeview Instruments AB, Vänge, Sweden) and the equilibrium dissociation constant (K_D_) was calculated. The interaction heterogeneity was evaluated by Interaction Map software (Ridgeview Diagnostics AB, Uppsala, Sweden).

### 4.8. In Vitro Proliferation Assay

The effect of Ec1–LoPE on cell proliferation was studied using EpCAM-expressing BxPC3 and Panc-1 cells. The cells were seeded in 96-well plates at a density of 2000 cells/well and incubated in a humidified incubator overnight to allow attachment. The next day, a serial dilution of Ec1–LoPE (0–1000 nM) in a culture medium containing 4% FBS was prepared and added to the cells (n = 5). The plates were incubated in a humidified incubator for 72 h. To test the specificity of the cytotoxic action of Ec1–LoPE, one group of cells was incubated with Ec1 (1000 nM) for 10 min before the addition of Ec1–LoPE (10 nM).

The inhibition of proliferation of BxPC3 cells by MM-121 in vitro was studied previously by Leitao et al. [36]. To evaluate the combined effect of Ec1–LoPE and MM-121 on the proliferation of EpCAM- and HER3-expressing BxPC3 cells, on day one, six concentrations of MM-121 (0, 1, 5, 10, 50, and 100 nM) in the presence of heregulin (4 nM) were prepared in culture medium containing 4% FBS and added to the cells. The plates were incubated in a humidified incubator for 48 h. On day three, the medium was exchanged with a solution containing Ec1–LoPE (0–1000 nM) and MM-121 (0–100 nM) in the presence of heregulin (4 nM) in a culture medium containing 4% FBS. The plates were incubated in a humidified incubator for additional 72 h.

Cell viability was measured using cell counting kit-8 (CCK-8; Sigma-Aldrich, St. Louis, MO, USA) according to the manufacturer’s protocol. Briefly, on the day of measurement the medium was replaced with fresh medium (100 μL) and CCK8 solution (10 μL) was added to each well, followed by incubation for 1–3 h. The absorbance (OD value) was measured at 450 nm for cell viability and at 655 nm for background. The values from wells containing cells in medium only (no Ec1–LoPE or MM-121) were used as the 100% viability control. The values from wells without cells were used as the 0% viability control. The relative viability was analyzed by GraphPad Prism (version 9.4.0; GraphPad Software, La Jolla, CA, USA) using a log(inhibitor) vs. response-variable slope (four parameters) model providing half-maximal inhibitory concentration (IC_50_) values.

The effect of Ec1–LoPE and MM-121 combination on the viability of BxPC3 cells was analyzed using the web application SynergyFinder 3.0 [67,68]. In the SynergyFinder web application, to evaluate the potential synergy of drugs, the observed combination responses were compared with expected combination responses calculated using combination scoring models. Based on the deviation of observed and expected combination responses, the drug combination was classified as synergistic (combination effect was higher than expected) or as antagonistic (combination effect was lower than expected). Highest single agent (HSA), Bliss, and Zero interaction potency (ZIP) reference models were used to calculate synergy scores. The has model is based on the assumption that the expected combination effect would equal to the maximum of the single drug responses; the Bliss model is based on the assumption that the expected combination effect can be calculated for two drugs that act independently; and the ZIP model captures the drug interaction by comparing the change in the potency of the dose–response curves between individual drugs and their combinations [69].

### 4.9. Experimental Therapy

The animal experiments were planned and performed following Swedish national legislation on the protection of laboratory animals. The experiments were approved by the local ethical committee for animal research in Uppsala, Sweden (permit 5.8.18-11931/2020, approved 28 August 2020).

Thirty nine female Balb/c nu/nu mice were subcutaneously (s.c.) implanted on the abdomen with a 5 × 10^6^ BxPC-3 cells (100 µL in culture medium), and one week later (day 0) the mice were randomized into four groups: MM-121 antibody monotherapy, targeted toxin Ec1–LoPE monotherapy, MM-121 and Ec1–LoPE combined therapy, and vehicle injection control. At the start of the experimental therapy, the average tumor volume was 97 ± 24 mm^3^ and the average mouse weight was 19 ± 1 g.

In the antibody monotherapy group, the mice received an intraperitoneal injection of MM-121 antibody (2.1 nmol, 300 µg, 16 mg/kg in 100 μL of 0.9% NaCl) every third day between day 0 (treatment start) and day 28. In the Ec1–LoPE monotherapy group, the animals received two cycles of therapy. During each cycle, Ec1–LoPE was intravenously (i.v.) injected four times, every second day. The first cycle started on day 0, the second cycle started on day 21, and the injected dose was 0.46 nmol (20 µg, 1 mg/kg in 100 μL of 0.9% NaCl). In the combined therapy group, the injections of MM-121 antibody and Ec1–LoPE targeted toxin were performed at the same dosage and with the same schedule as in the monotherapy groups. In the control group, mice were i.v. injected with the vehicle (100 μL of 0.9% NaCl) on the same days as in the combined therapy group.

The tumor dimensions were measured using a digital caliper for the largest longitudinal (length) and transverse (width) diameter twice per week. The tumor volume was calculated using the formula: tumor volume = 1/2(length × width^2^). The mice’s status was monitored twice per week. The mice were euthanized when they reached a predetermined humane endpoint (the subcutaneous tumor volume exceeded 1000 mm^3^; bleeding sores on the tumor were observed; 15% overall weight loss or 10% weight loss within one week). According to the ethical permit, the study endpoint was 90 days after the treatment started. When the mice were sacrificed, the livers and kidneys were excised, kept in formalin for 24 h, and thereafter stored in ethanol. The tissue samples were embedded in paraffin, sectioned (3–4 µm thickness), and stained with hematoxylin and eosin. The stained samples were investigated for histopathologic changes by a veterinary pathologist at a veterinary medicine laboratory (BioVet AB, Sollentuna, Sweden).

### 4.10. Statistical Analysis

GraphPad Prism (version 9.4.0, GraphPad Software, La Jolla, CA, USA) was used to generate plots and perform statistical analysis. Data are presented as means ± SD (* *p* < 0.05, ** *p* < 0.01, *** *p* < 0.001, **** *p* < 0.001 and ns = not statistically different). Student’s *t*-test was used for comparisons between two different groups. For comparisons between multiple groups, analysis of variance (ANOVA) with Bonferroni’s post hoc multiple comparisons test was used.

## 5. Conclusions

In conclusion, the co-targeting of EpCAM and HER3 using Ec1–LoPE and MM-121 is feasible. The combination of Ec1–LoPE and MM-121 provided a synergistic cytotoxic effect on BxPC3 cells in vitro. The experimental therapy study using Ec1–LoPE and MM-121 in mice bearing BxPC3 xenografts demonstrated the feasibility of the therapy.

## Figures and Tables

**Table 1:** Equilibrium dissociation constants (K_D_) for the interaction of [^99m^Tc]Tc(CO)_3_-Ec1–LoPE with EpCAM-expressing BxPC3 cells. The data are from four samples (n = 4).

Cell Line	K_D1_ (nM)	Weight (%)	K_D2_ (nM)	Weight (%)
BxPC3 (n = 4)	1.03 ± 0.05	31 ± 8	27 ± 9	46 ± 4

## Data Availability

The data generated during the current study are available from the corresponding author upon reasonable request.

## Supplementary Materials

The following supporting information can be downloaded at: https://www.mdpi.com/article/10.3390/ijms24032838/s1.

## References

[1] Lambert A., Schwarz L., Borbath I., Henry A., Van Laethem J.L., Malka D., Ducreux M., Conroy T. (2019). An update on treatment options for pancreatic adenocarcinoma. Ther. Adv. Med. Oncol..

[2] Long J., Luo G.P., Xiao Z.W., Liu Z.Q., Guo M., Liu L., Liu C., Xu J., Gao Y.T., Zheng Y. (2014). Cancer statistics: Current diagnosis and treatment of pancreatic cancer in Shanghai, China. Cancer Lett..

[3] Burkhardt C., Bühler L., Viertl D., Stora T. (2021). New Isotopes for the Treatment of Pancreatic Cancer in Collaboration with CERN: A Mini Review. Front. Med..

[4] Ansari D., Tingstedt B., Andersson B., Holmquist F., Sturesson C., Williamsson C., Sasor A., Borg D., Bauden M., Andersson R. (2016). Pancreatic cancer: Yesterday, today and tomorrow. Future Oncol..

[5] Grant T.J., Hua K., Singh A. (2016). Molecular Pathogenesis of Pancreatic Cancer. Prog. Mol. Biol. Transl. Sci..

[6] Tran N.H., Bekaii-Saab T. (2021). Optimizing Chemotherapy Choice in the Treatment of Advanced Pancreatic Cancer-It Is Complicated. JAMA Netw. Open.

[7] Ducreux M., Cuhna A.S., Caramella C., Hollebecque A., Burtin P., Goéré D., Seufferlein T., Haustermans K., Van Laethem J.L., Conroy T. (2015). Cancer of the pancreas: ESMO Clinical Practice Guidelines for diagnosis, treatment and follow-up. Ann. Oncol..

[8] Moore M.J., Goldstein D., Hamm J., Figer A., Hecht J.R., Gallinger S., Au H.J., Murawa P., Walde D., Wolff R.A. (2007). Erlotinib plus gemcitabine compared with gemcitabine alone in patients with advanced pancreatic cancer: A phase III trial of the National Cancer Institute of Canada Clinical Trials Group. J. Clin. Oncol..

[9] Kindler H.L., Hammel P., Reni M., Van Cutsem E., Macarulla T., Hall M.J., Park J.O., Hochhauser D., Arnold D., Oh D.Y. (2022). Overall Survival Results from the POLO Trial: A Phase III Study of Active Maintenance Olaparib Versus Placebo for Germline BRCA-Mutated Metastatic Pancreatic Cancer. J. Clin. Oncol..

[10] Kindler H.L., Niedzwiecki D., Hollis D., Sutherland S., Schrag D., Hurwitz H., Innocenti F., Mulcahy M.F., O’Reilly E., Wozniak T.F. (2010). Gemcitabine plus bevacizumab compared with gemcitabine plus placebo in patients with advanced pancreatic cancer: Phase III trial of the cancer and leukemia group B (CALGB 80303). J. Clin. Oncol..

[11] Forster T., Huettner F.J., Springfeld C., Loehr M., Kalkum E., Hackbusch M., Hackert T., Diener M.K., Probst P. (2020). Cetuximab in Pancreatic Cancer Therapy: A Systematic Review and Meta-Analysis. Oncology.

[12] Miller A.L., Garcia P.L., Yoon K.J. (2020). Developing effective combination therapy for pancreatic cancer: An overview. Pharmacol. Res..

[13] Burris H.A., Moore M.J., Andersen J., Green M.R., Rothenberg M.L., Modiano M.R., Cripps M.C., Portenoy R.K., Storniolo A.M., Tarassoff P. (1997). Improvements in survival and clinical benefit with gemcitabine as first-line therapy for patients with advanced pancreas cancer: A randomized trial. J. Clin. Oncol..

[14] Conroy T., Desseigne F., Ychou M., Bouché O., Guimbaud R., Bécouarn Y., Adenis A., Raoul J.L., Gourgou-Bourgade S., de la Fouchardière C. (2011). FOLFIRINOX versus gemcitabine for metastatic pancreatic cancer. N. Engl. J. Med..

[15] Peixoto R.D., Ho M., Renouf D.J., Lim H.J., Gill S., Ruan J.Y., Cheung W.Y. (2017). Eligibility of Metastatic Pancreatic Cancer Patients for First-Line Palliative Intent nab-Paclitaxel Plus Gemcitabine Versus FOLFIRINOX. Am. J. Clin. Oncol..

[16] Hynes N.E., MacDonald G. (2009). ErbB receptors and signaling pathways in cancer. Curr. Opin. Cell Biol..

[17] Koumakpayi I.H., Diallo J.S., Le Page C., Lessard L., Gleave M., Bégin L.R., Mes-Masson A.M., Saad F. (2006). Expression and nuclear localization of ErbB3 in prostate cancer. Clin. Cancer Res..

[18] Liles J.S., Arnoletti J.P., Tzeng C.W., Howard J.H., Kossenkov A.V., Kulesza P., Heslin M.J., Frolov A. (2010). ErbB3 expression promotes tumorigenesis in pancreatic adenocarcinoma. Cancer Biol. Ther..

[19] Ocana A., Vera-Badillo F., Seruga B., Templeton A., Pandiella A., Amir E. (2013). HER3 overexpression and survival in solid tumors: A meta-analysis. J. Natl. Cancer Inst..

[20] Scartozzi M., Mandolesi A., Giampieri R., Bittoni A., Pierantoni C., Zaniboni A., Galizia E., Giustini L., Silva R.R., Bisonni R. (2011). The role of HER-3 expression in the prediction of clinical outcome for advanced colorectal cancer patients receiving irinotecan and cetuximab. Oncologist.

[21] Siegfried J.M., Lin Y., Diergaarde B., Lin H.M., Dacic S., Pennathur A., Weissfeld J.L., Romkes M., Nukui T., Stabile L.P. (2015). Expression of PAM50 Genes in Lung Cancer: Evidence that Interactions between Hormone Receptors and HER2/HER3 Contribute to Poor Outcome. Neoplasia.

[22] Tanner B., Hasenclever D., Stern K., Schormann W., Bezler M., Hermes M., Brulport M., Bauer A., Schiffer I.B., Gebhard S. (2006). ErbB-3 predicts survival in ovarian cancer. J. Clin. Oncol..

[23] Bittoni A., Mandolesi A., Andrikou K., Santoni M., Alfonsi S., Lanese A., Loretelli C., Pellei C., Piva F., Scarpelli M. (2015). HER family receptor expression and prognosis in pancreatic cancer. Int. J. Biol. Markers.

[24] Frolov A., Schuller K., Tzeng C.W., Cannon E.E., Ku B.C., Howard J.H., Vickers S.M., Heslin M.J., Buchsbaum D.J., Arnoletti J.P. (2007). ErbB3 expression and dimerization with EGFR influence pancreatic cancer cell sensitivity to erlotinib. Cancer Biol. Ther..

[25] Schoeberl B., Kudla A., Masson K., Kalra A., Curley M., Finn G., Pace E., Harms B., Kim J., Kearns J. (2017). Systems biology driving drug development: From design to the clinical testing of the anti-ErbB3 antibody seribantumab (MM-121). NPJ Syst. Biol. Appl..

[26] ClinicalTrials.gov (2005). Phase 2 Trial of Seribantumab Plus Fulvestrant in Postmenopausal Women with Metastatic Breast Cancer (SHERBOC).

[27] Jacob W., James I., Hasmann M., Weisser M. (2018). Clinical development of HER3-targeting monoclonal antibodies: Perils and progress. Cancer Treat. Rev..

[28] Sequist L.V., Gray J.E., Harb W.A., Lopez-Chavez A., Doebele R.C., Modiano M.R., Jackman D.M., Baggstrom M.Q., Atmaca A., Felip E. (2019). Randomized Phase II Trial of Seribantumab in Combination with Erlotinib in Patients with EGFR Wild-Type Non-Small Cell Lung Cancer. Oncologist.

[29] Larimer B.M., Phelan N., Wehrenberg-Klee E., Mahmood U. (2018). Phage Display Selection, In Vitro Characterization, and Correlative PET Imaging of a Novel HER3 Peptide. Mol. Imaging Biol..

[30] Lockhart A.C., Liu Y., Dehdashti F., Laforest R., Picus J., Frye J., Trull L., Belanger S., Desai M., Mahmood S. (2016). Phase 1 Evaluation of [(64)Cu]DOTA-Patritumab to Assess Dosimetry, Apparent Receptor Occupancy, and Safety in Subjects with Advanced Solid Tumors. Mol. Imaging Biol..

[31] Menke-van der Houven van Oordt C.W., McGeoch A., Bergstrom M., McSherry I., Smith D.A., Cleveland M., Al-Azzam W., Chen L., Verheul H., Hoekstra O.S. (2019). Immuno-PET Imaging to Assess Target Engagement: Experience from 89Zr-Anti-HER3 mAb (GSK2849330) in Patients with Solid Tumors. J. Nucl. Med..

[32] Rinne S.S., Leitao C.D., Abouzayed A., Vorobyeva A., Tolmachev V., Ståhl S., Löfblom J., Orlova A. (2021). HER3 PET Imaging: 68Ga-Labeled Affibody Molecules Provide Superior HER3 Contrast to 89Zr-Labeled Antibody and Antibody-Fragment-Based Tracers. Cancers.

[33] Pool M., Kol A., de Jong S., de Vries E.G.E., Lub-de Hooge M.N., van Scheltinga A.G.T.T. (2017). 89Zr-mAb3481 PET for HER3 tumor status assessment during lapatinib treatment. MAbs.

[34] van Scheltinga A.G.T., Lub-de Hooge M.N., Abiraj K., Schröder C.P., Pot L., Bossenmaier B., Thomas M., Hölzlwimmer G., Friess T., Kosterink J.G. (2014). ImmunoPET and biodistribution with human epidermal growth factor receptor 3 targeting antibody ⁸⁹Zr-RG7116. MAbs.

[35] Warnders F.J., Terwisscha van Scheltinga A.G.T., Knuehl C., van Roy M., de Vries E.F.J., Kosterink J.G.W., de Vries E.G.E., Lub-de Hooge M.N. (2017). Human Epidermal Growth Factor Receptor 3-Specific Tumor Uptake and Biodistribution of 89Zr-MSB0010853 Visualized by Real-Time and Noninvasive PET Imaging. J. Nucl. Med..

[36] Leitao C.D., Rinne S.S., Altai M., Vorontsova O., Dunås F., Jonasson P., Tolmachev V., Löfblom J., Ståhl S., Orlova A. (2020). Evaluating the Therapeutic Efficacy of Mono- and Bivalent Affibody-Based Fusion Proteins Targeting HER3 in a Pancreatic Cancer Xenograft Model. Pharmaceutics.

[37] Fagotto F., Aslemarz A. (2020). EpCAM cellular functions in adhesion and migration, and potential impact on invasion: A critical review. Biochim. Biophys. Acta Rev. Cancer.

[38] Gires O., Pan M., Schinke H., Canis M., Baeuerle P.A. (2020). Expression and function of epithelial cell adhesion molecule EpCAM: Where are we after 40 years?. Cancer Metastasis Rev..

[39] Fong D., Steurer M., Obrist P., Barbieri V., Margreiter R., Amberger A., Laimer K., Gastl G., Tzankov A., Spizzo G. (2008). Ep-CAM expression in pancreatic and ampullary carcinomas: Frequency and prognostic relevance. J. Clin. Pathol..

[40] Spizzo G., Fong D., Wurm M., Ensinger C., Obrist P., Hofer C., Mazzoleni G., Gastl G., Went P. (2011). EpCAM expression in primary tumour tissues and metastases: An immunohistochemical analysis. J. Clin. Pathol..

[41] Balzar M., Winter M.J., de Boer C.J., Litvinov S.V. (1999). The biology of the 17-1A antigen (Ep-CAM). J. Mol. Med..

[42] Di Paolo C., Willuda J., Kubetzko S., Lauffer I., Tschudi D., Waibel R., Plückthun A., Stahel R.A., Zangemeister-Wittke U. (2003). A recombinant immunotoxin derived from a humanized epithelial cell adhesion molecule-specific single-chain antibody fragment has potent and selective antitumor activity. Clin. Cancer Res..

[43] Hussain S., Plückthun A., Allen T.M., Zangemeister-Wittke U. (2006). Chemosensitization of carcinoma cells using epithelial cell adhesion molecule-targeted liposomal antisense against bcl-2/bcl-xL. Mol. Cancer Ther..

[44] Hussain S., Plückthun A., Allen T.M., Zangemeister-Wittke U. (2007). Antitumor activity of an epithelial cell adhesion molecule targeted nanovesicular drug delivery system. Mol. Cancer Ther..

[45] Macdonald J., Henri J., Roy K., Hays E., Bauer M., Veedu R.N., Pouliot N., Shigdar S. (2018). EpCAM Immunotherapy versus Specific Targeted Delivery of Drugs. Cancers.

[46] Frøysnes I.S., Andersson Y., Larsen S.G., Davidson B., Øien J.T., Julsrud L., Fodstad Ø., Dueland S., Flatmark K. (2021). ImmunoPeCa trial: Long-term outcome following intraperitoneal MOC31PE immunotoxin treatment in colorectal peritoneal metastasis. Eur. J. Surg. Oncol..

[47] Shramova E., Proshkina G., Shipunova V., Ryabova A., Kamyshinsky R., Konevega A., Schulga A., Konovalova E., Telegin G., Deyev S. (2020). Dual Targeting of Cancer Cells with DARPin-Based Toxins for Overcoming Tumor Escape. Cancers.

[48] Xu T., Vorobyeva A., Schulga A., Konovalova E., Vorontsova O., Ding H., Gräslund T., Tashireva L.A., Orlova A., Tolmachev V. (2021). Imaging-Guided Therapy Simultaneously Targeting HER2 and EpCAM with Trastuzumab and EpCAM-Directed Toxin Provides Additive Effect in Ovarian Cancer Model. Cancers.

[49] Xu T., Liu Y., Schulga A., Konovalova E., Deyev S.M., Tolmachev V., Vorobyeva A. (2022). Epithelial cell adhesion molecule-targeting designed ankyrin repeat protein-toxin fusion Ec1-LoPE exhibits potent cytotoxic action in prostate cancer cells. Oncol. Rep..

[50] Plückthun A. (2015). Designed ankyrin repeat proteins (DARPins): Binding proteins for research, diagnostics, and therapy. Annu. Rev. Pharmacol. Toxicol..

[51] Sokolova E., Proshkina G., Kutova O., Shilova O., Ryabova A., Schulga A., Stremovskiy O., Zdobnova T., Balalaeva I., Deyev S. (2016). Recombinant targeted toxin based on HER2-specific DARPin possesses a strong selective cytotoxic effect in vitro and a potent antitumor activity in vivo. J. Control. Release.

[52] Sokolova E.A., Shilova O.N., Kiseleva D.V., Schulga A.A., Balalaeva I.V., Deyev S.M. (2019). HER2-Specific Targeted Toxin DARPin-LoPE: Immunogenicity and Antitumor Effect on Intraperitoneal Ovarian Cancer Xenograft Model. Int. J. Mol. Sci..

[53] Vorobyeva A., Konovalova E., Xu T., Schulga A., Altai M., Garousi J., Rinne S.S., Orlova A., Tolmachev V., Deyev S. (2020). Feasibility of Imaging EpCAM Expression in Ovarian Cancer Using Radiolabeled DARPin Ec1. Int. J. Mol. Sci..

[54] Liu W., Onda M., Lee B., Kreitman R.J., Hassan R., Xiang L., Pastan I. (2012). Recombinant immunotoxin engineered for low immunogenicity and antigenicity by identifying and silencing human B-cell epitopes. Proc. Natl. Acad. Sci. USA.

[55] Lopez J.S., Banerji U. (2017). Combine and conquer: Challenges for targeted therapy combinations in early phase trials. Nat. Rev. Clin. Oncol..

[56] Nishimoto A. (2022). Effective combinations of anti-cancer and targeted drugs for pancreatic cancer treatment. World J. Gastroenterol..

[57] Weldon J.E., Pastan I. (2011). A guide to taming a toxin—Recombinant immunotoxins constructed from Pseudomonas exotoxin A for the treatment of cancer. FEBS J..

[58] Schoeberl B., Faber A.C., Li D., Liang M.C., Crosby K., Onsum M., Burenkova O., Pace E., Walton Z., Nie L. (2010). An ErbB3 antibody, MM-121, is active in cancers with ligand-dependent activation. Cancer Res..

[59] Orlova A., Nilsson F.Y., Wikman M., Widström C., Ståhl S., Carlsson J., Tolmachev V. (2006). Comparative in vivo evaluation of technetium and iodine labels on an anti-HER2 affibody for single-photon imaging of HER2 expression in tumors. J. Nucl. Med..

[60] Stefan N., Martin-Killias P., Wyss-Stoeckle S., Honegger A., Zangemeister-Wittke U., Plückthun A. (2011). DARPins recognizing the tumor-associated antigen EpCAM selected by phage and ribosome display and engineered for multivalency. J. Mol. Biol..

[61] Deyev S.M., Vorobyeva A., Schulga A., Abouzayed A., Günther T., Garousi J., Konovalova E., Ding H., Gräslund T., Orlova A. (2020). Effect of a radiolabel biochemical nature on tumor-targeting properties of EpCAM-binding engineered scaffold protein DARPin Ec1. Int. J. Biol. Macromol..

[62] Meng Y., Xu B.Q., Fu Z.G., Wu B., Xu B., Chen Z.N., Li L. (2015). Cytoplasmic EpCAM over-expression is associated with favorable clinical outcomes in pancreatic cancer patients with Hepatitis B virus negative infection. Int. J. Clin. Exp. Med..

[63] Lund K., Bostad M., Skarpen E., Braunagel M., Kiprijanov S., Krauss S., Duncan A., Høgset A., Selbo P.K. (2014). The novel EpCAM-targeting monoclonal antibody 3-17I linked to saporin is highly cytotoxic after photochemical internalization in breast, pancreas and colon cancer cell lines. MAbs.

[64] Rosestedt M., Andersson K.G., Mitran B., Tolmachev V., Löfblom J., Orlova A., Ståhl S. (2015). Affibody-mediated PET imaging of HER3 expression in malignant tumours. Sci. Rep..

[65] Teixidó C., Marés R., Aracil M., Cajal S.R.Y., Hernández-Losa J. (2013). Epithelial-Mesenchymal Transition Markers and HER3 Expression Are Predictors of Elisidepsin Treatment Response in Breast and Pancreatic Cancer Cell Lines. PLoS ONE.

[66] Wållberg H., Orlova A. (2008). Slow internalization of anti-HER2 synthetic affibody monomer 111In-DOTA-ZHER2:342-pep2: Implications for development of labeled tracers. Cancer Biother. Radiopharm..

[67] Ianevski A., Giri A.K., Aittokallio T. (2022). SynergyFinder 3.0: An interactive analysis and consensus interpretation of multi-drug synergies across multiple samples. Nucleic Acids Res..

[68] Ianevski A. SynergyFinder Web Application (Version 3.0): An Interactive Analysis of Multi-Drug Combination Data.

[69] Yadav B., Wennerberg K., Aittokallio T., Tang J. (2015). Searching for Drug Synergy in Complex Dose-Response Landscapes Using an Interaction Potency Model. Comput. Struct. Biotechnol. J..

