# Supplementary material for: Feasibility of Co-Targeting HER3 and EpCAM Using Seribantumab and DARPin–Toxin Fusion in a Pancreatic Cancer Xenograft Model
Source: Int J Mol Sci. 2023 Feb 2;24(3):2838. doi: 10.3390/ijms24032838 (PMC9917732; doi:10.3390/ijms24032838)
Supplement: Supplementary file 1 [file ijms-24-02838-s001.zip › ijms-2065218-supplementary.pdf]

Supplementary Information

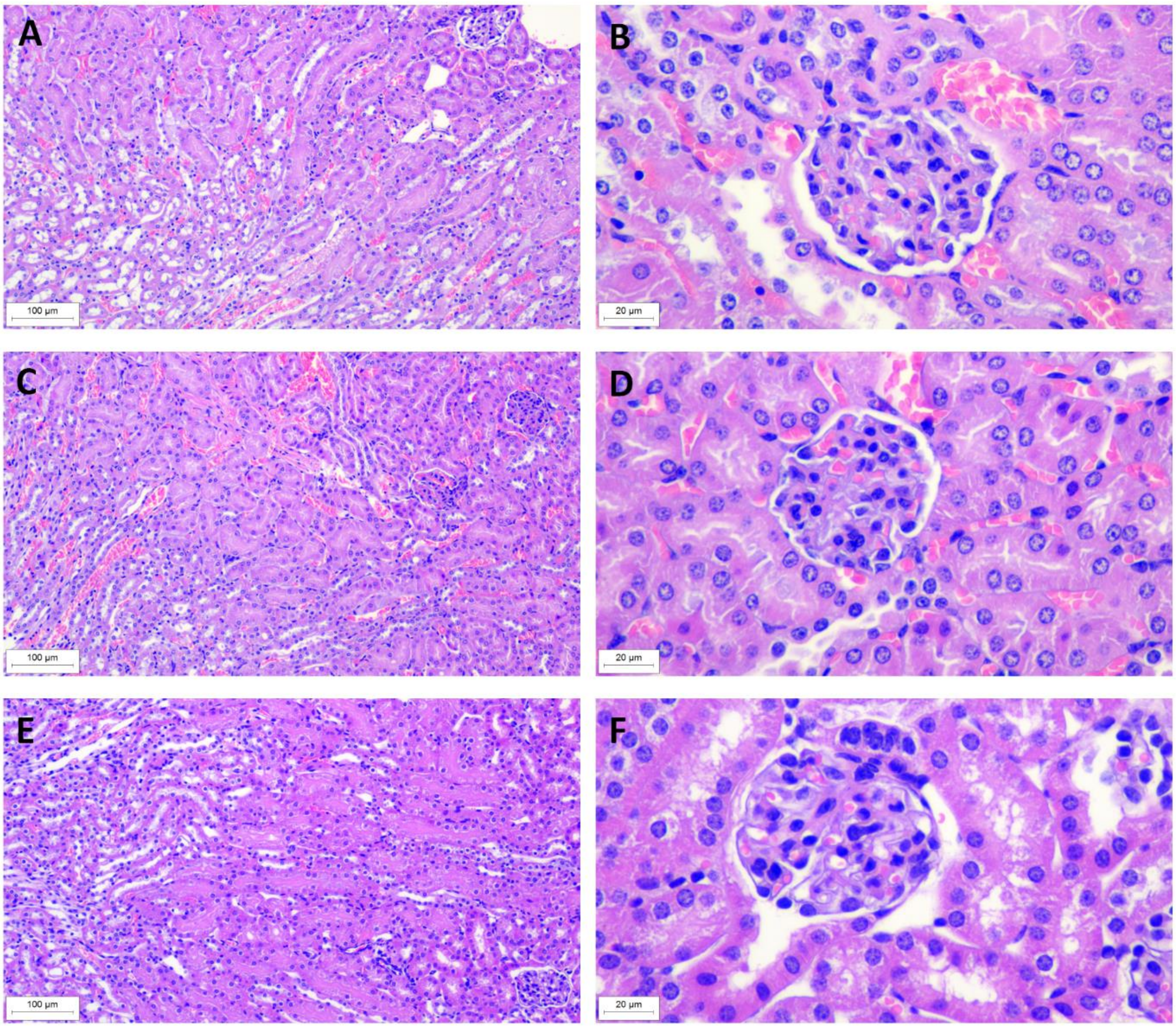

Figure S1. Representative images of pathological examination of kidneys. Hematoxylin and eosin stain, 100x and 400x of kidney in a mouse from (A, B) Ec1-LoPE and MM-121 combination group A, (C, D) Ec1-LoPE monotherapy group B and (E, F) vehicle control group D.

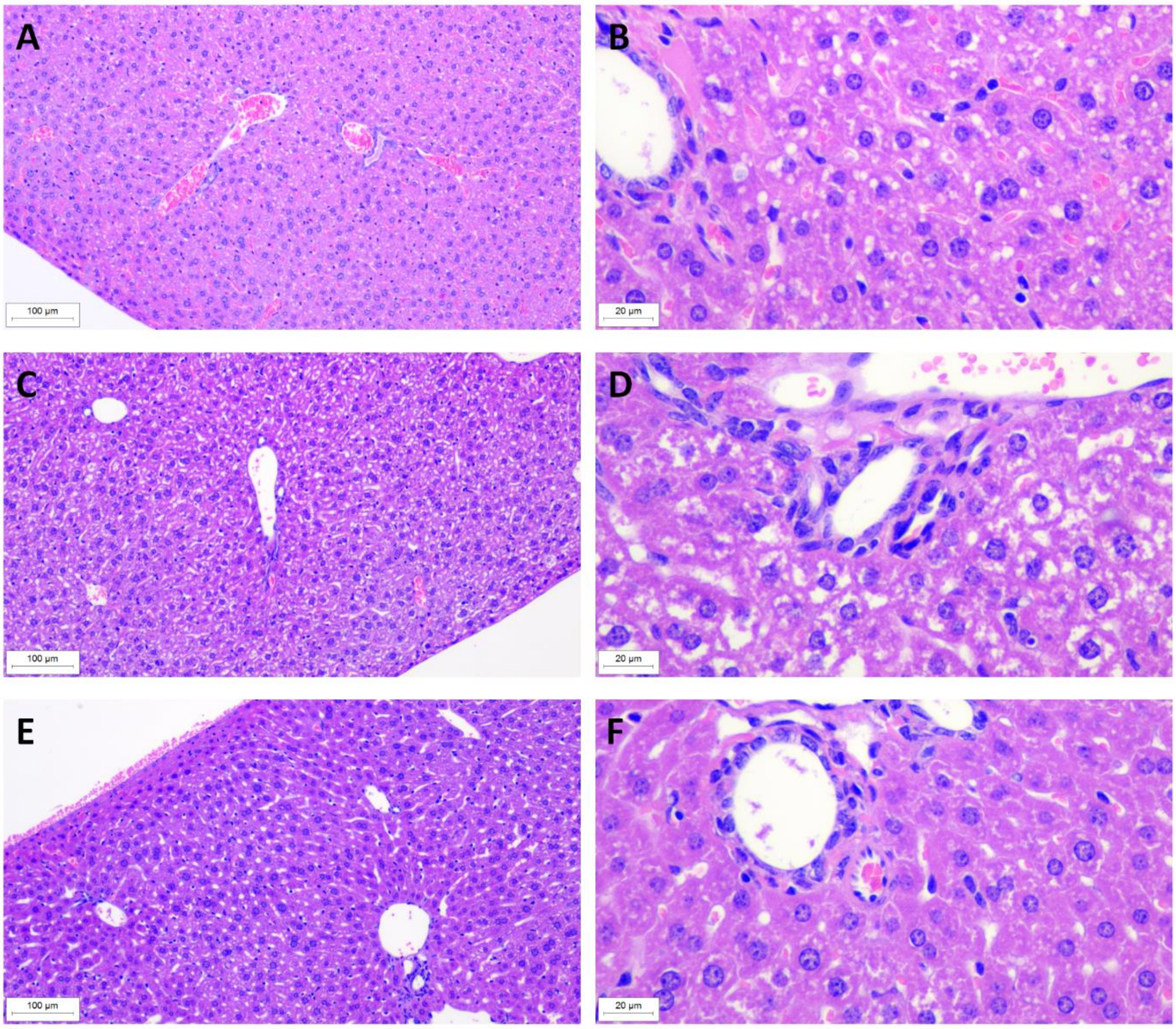

Figure S2. Representative images of pathological examination of liver. Hematoxylin and eosin stain, 100x and 400x of liver in a mouse from (A, B) Ec1-LoPE and MM-121 combination group A, (C, D) Ec1-LoPE monotherapy group B and (E, F) vehicle control group D.

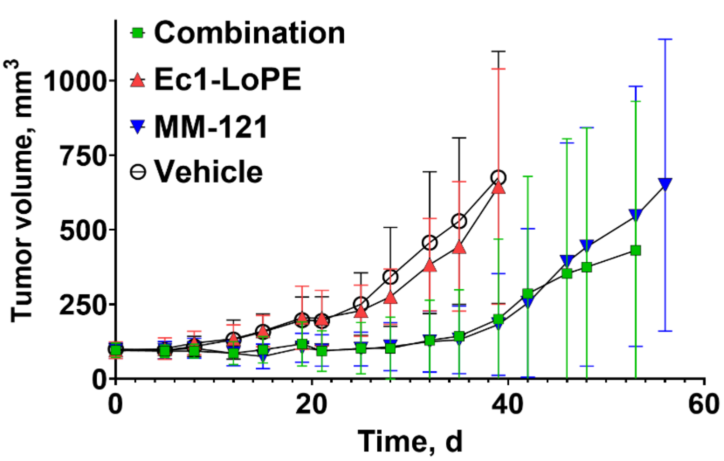

Figure S3. The average tumor volume growth curves in BALB/c nu/nu mice bearing BxPC3 xenografts from four groups (n = 9-10) receiving combination of Ec1-LoPE and MM-121, Ec1-LoPE only, MM-121 only and vehicle (0.9% NaCl). The data are presented as average ± standard deviation (SD). The curves were drawn until 30-33% of the mice in a group were euthanized.

Table S1. Individual tumor volume (mm^3) during experimental therapy in BALB/C nu/nu mice bearing BxPC-3 xenografts receiving combination of Ec1-LoPE and MM-121 (group A); Ec1-LoPE alone (group B); MM-121 alone (group C); vehicle (0.9% NaCl, group D).

Day	0	5	8	12	15	19	21	25	28	32	35	39	42	46	48	53	56	59	63	67	70	73	76	80	84	87	90
A1	107	98	101	137	73	71	34	30	18	10	8	7	14	17	26	61	63	134	181	222	282	393	381	407	500	559	554
A2	79	104	69	55	65	88	62	40	23	35	54	30	46	36	31	39	37	43	59	42	43	51	51	56	42	39	42
A3	96	99	107	120	159	189	119	95	88	178	164	108	227	352	310	456	527	690	1040								
A4	99	97	107	138	163	250	210	259	318	414	486	840	1164														
A5	87	61	70	55	41	57	38	40	18	14	29	30	40	51	51	58	78	129	191	279	349	441	508	696	747		
A6	83	86	95	82	115	68	53	69	34	26	30	76	99	117	96	104	100	96	133	183	335	511	657	764	1122		
A7	77	85	82	73	79	121	111	126	150	189	189	262	339	597	811	1122											
A8	83	81	71	41	108	74	76	105	146	206	157	143	66	62	124	170	169	234	248	360	472						
A9	91	85	85	45	44	39	21	17	11	6	6	23	48	25	27	27	37	32	50	32	37	36	0	0	23	17	23
A10	158	139	143	117	129	214	206	244	218	214	305	484	814	1107													
B1	122	125	153	183	266	475	422	301	352	387	415	572	983														
B2	136	142	187	196	237	286	261	366	420	513	626	1142															
B3	59	52	76	92	111	169	121	199	222	350	279	353	667	1026													
B4	76	74	89	100	136	128	154	158	227	344	418	606	892	1186													
B5	119	101	128	182	161	193	240	316	396	724	833	1181															
B6	95	83	124	135	115	204	232	190	197	228	239	326	369	884	1181												
B7	123	143	126	142	167	138	105	118	171	303	215	200	319	528	626	824	1133										
B8	57	56	65	68	157	173	191	261	258	333	580	577	711	1004													
B9	81	93	90	83	112	138	124	114	175	182	197	267	352	404	569	776	1000										
B10	93	146	159	164	138	141	181	259	334	464	635	1217															
C1	42	61	86	62	46	69	68	104	143	124	149	143	280	445	546	716	876	1097									
C2	105	72	76	69	62	76	68	78	87	98	145	159	276	410	488	774	984										
C3	64	57	71	45	48	67	56	73	63	35	35	49	32	49	39	24	31	33	14	39	33	33	38	34	57	0	0
C4	114	126	152	118	112	150	130	117	119	97	87	111	134	237	381	716	1042										
C5	82	76	68	68	66	93	83	95	120	138	152	233	276	347	528	787	1044										
C6	106	115	127	113	140	161	165	198	289	317	358	530	777	1139													
C7	131	140	108	64	23	35	23	8	0	0	0	30	30	25	39	34	54	48	53	66	64	45	39	54	51	43	37
C8	108	83	64	42	33	71	53	36	9	6	6	19	25	59	28	26	35	30	40	22	35	30	30	25	34	0	34
C9	87	108	135	95	90	156	121	124	112	190	107	121	138	151	186	182	238	220	222	493	683	1007					
C10	109	122	138	177	132	164	185	167	133	242	267	428	575	1048													
D1	111	119	126	104	122	115	92	161	194	205	242	241	388	470	687												
D2	77	48	55	50	62	83	113	146	184	320	319	384	574	850	1050												
D3	84	86	104	127	149	223	210	272	407	563	628	750	1283														
D4	135	84	102	88	132	189	200	295	447	603	671	1062															
D5	83	97	119	133	158	286	248	368	619	840	927	912	1280														
D6	96	80	71	95	170	185	194	205	289	310	357	260															
D7	135	138	164	268	221	235	192	171	206	292	334	582	842	1166													
D8	101	129	142	204	274	317	364	452	537	753	969	1498															
D9	75	82	93	113	130	137	134	183	192	225	314	384	591	657	934												
